# Digital Scholarship – rethinking educational scholarship in the digital world

**DOI:** 10.15694/mep.2019.000085.1

**Published:** 2019-04-15

**Authors:** Poh-Sun Goh, John Sandars

**Affiliations:** 1 National University of Singapore; 2 Edge Hill University Medical School

**Keywords:** Digital Scholarship, Scholarship of Teaching and Learning, Technology Enhanced Learning

## Abstract

This article was migrated. The article was marked as recommended.

Medical education is increasingly becoming a digital world, with a range of new technologies that are transforming and challenging our current activities as a medical educator. The purpose of this article is to highlight how technology not only supports teaching and learning but also offers new opportunities for demonstrating the educational scholarship of medical educators.

## Introduction

The foundation of educational scholarship that has been widely adopted by medical educators has a focus on the engagement and public demonstration of scholarly educational activities in research and teaching ( Boyer, 1990; Glassick, 2000). Educators are expected to engage with the scholarship of discovery to identify new knowledge through research and to also disseminate and apply the knowledge to practice. This is usually demonstrated by a limited range of scholarly outputs, especially publications and conference presentations. Educators are also expected to engage with the scholarship of teaching to identify the impact of their teaching activities on the learning of their students. This is usually demonstrated by student satisfaction surveys and achievement of specific aspects of student performance, such as examination grades. Educators can collate all of this information for various purposes, especially job appraisals and promotion committees, and this is usually in the form of a paper based portfolio.

## Technology transforms educational scholarship

Over the last few years, there has been increasing awareness of how engagement and demonstration of scholarly educational research and teaching activities have been transformed by a variety of new technologies ( Raffaghelli *et al.*, 2016; Raffaghelli, 2017). There are three main areas in which technologies have led transformation:

### (a) Open access publication and non-peer reviewed outputs

The rise in open access publishing has been almost exponential, with numerous opportunities for scholarly outputs to be published without cost to the reader. In contrast, most established academic journals require readers to pay a subscription or be a member of a subscribing organization and this limits wider access by readers, especially in low resource settings, and the potential wider impact of any outputs. Some open access journals have a formal peer review process prior to publication, such as BMC Medical Education, but others have adopted a post publication peer review process, such as MedEd Publish. Non-peer reviewed scholarly outputs, including research and teaching, are frequently disseminated through conference proceedings, but many conferences also have an element of peer review.

### (b) Social Media

There is an increasing number and variety of social media, such as Facebook, WhatsApp, Instagram, and Twitter, that are freely accessible from any online or mobile platform. Scholarly outputs from research and teaching can be quickly published and shared across the world but also scholarly activity in collaboration online networks and communities of practice can be developed ( Cabrera, Roy and Chisolm, 2018; Carrigan, 2017). For example multiple technologies can provide online digital content for medical education as part of FOAM (Free Open Access Meducation) and these include blogs, podcasts, videos and increasingly Virtual Reality (VR) and Augmented Reality (AR). The impact of FOAM is potentially significant in settings where training is limited ( Burkholder, Bellows and King, 2018).

### (c) Analytics

The use of digital analytics to measure various online activities has rapidly risen over the last few years. The number of views of open access publications and social media sites can be easily measured using online free tools such as Google Analytics, and established platforms like Almetrics and this data can provide an indicator of the interest and usefulness of the scholarly output. The use of digital analytics can also provide useful insights into both learning and the impact of teaching, which are essential to evidence the scholarship of teaching ( Goh and Sandars, 2018; Goh, 2017; Goh, 2016).

The use of technology to support educational scholarship has been called digital scholarship, although a more specific conceptual definition is blurred because of the diverse but specific application of the term to academic publications and library science, academic engagement in social media and digital humanities, with the archiving information for study ( Weller, 2011).

## Technology challenges educational scholarship

The main challenge to educational scholarship by the use of new technologies is the recognition of how the diverse types of scholarly engagement and output in research and teaching can be used for existing systems of appraisal and promotion.

Non-peer reviewed outputs are often considered to be of inferior quality compared with scholarly work that is peer reviewed but concerns have been expressed about the vagaries of the peer review process, specifically focusing on bias, though the validity of various forms of bias can be challenged ( Lee *et al*., 2013). There are many non-peer reviewed outputs that are now subject to a stream of peer feedback and author replies, either through the output websites or social media. For example, there are published peer reviews associated with FOAM content in blogs ( Thoma *et.al.*, 2015). This exchange is visible to all viewers and can be considered as rigorous as traditional peer review ( Stuntz and Clontz, 2016). There is also the added concern about non- reviewed content, such as FOAM, as the primary educational resource since it may not be balanced and comprehensive ( Stuntz and Clontz, 2016). This issue is likely to increasingly occur with the rise in this type of content and medical educators will need to change their role from creators of content to curators ( Goh, 2015).

The use of social media is also non-peer reviewed but there are now strong arguments that support social media as a legitimate form of educational scholarship, particularly when the use of social media can be assessed using the wider accepted guidelines for educational scholarship. Frameworks for assessing the educational scholarship of social media in medical education have recently been proposed by emergency physicians ( Thoma *et.al.*, 2015; Carrigan, 2017) and radiologists ( Cabrera, 2018).

Digital analytic metrics provide data on the number of views and links to published online content, which can be directly related to the size of the online audience (interest), and usefulness of the content (audience size, and recommendation). This data can be used to assess the impact of digital scholarship. An interesting approach to assessing the scholarship of teaching is the integration of teaching and learning analytics ( Sergis and Samson, 2017). Teaching analytics can analyse the design and delivery components of key teaching interventions by identifying explicit statements about how content is provided to learners and the methods of assessment. Learning analytics can offer additional data on the response of the learner to the teaching interventions. Reflection on the relationship and alignment between the teaching and learning analytic data can provide an essential tool for the assessment of the scholarship of teaching.

The process of building a portfolio of educational scholarship can be simplified using an electronic portfolio that collates and curates the different types of evidence, including weblinks to online publications and scholarly outputs, including social media, and teaching and learning analytics ( Olsson and Roxå, 2013; Olsson, Mårtensson and Roxå, 2010; Sonnenburg, von Hauff and Lemieux, 2017).

## Conclusion

Technologies can augment, enhance, extend and expand on the work of medical educators as medical educational scholars. The extension and expansion occurs through the use of open access dissemination and peer review, social media to build online networks and communities of practice, and the integration of teaching and learning analytics. Overall, digital scholarship facilitates and augments current activities in educational scholarship, with an essential focus on the reflective and creative approach to both the creation and presentation of the evidence required for the assessment of both the scholarship of research and the scholarship of teaching.

## Take Home Messages

Technologies can augment, enhance, extend and expand on the work of medical educators as medical educational scholars. The extension and expansion occurs through the use of open access dissemination and peer review, social media to build online networks and communities of practice , and the integration of teaching and learning analytics. Overall, digital scholarship facilitates and augments current activities in educational scholarship, with an essential focus on the reflective and creative approach to both the creation and presentation of the evidence required for the assessment of both the scholarship of research and the scholarship of teaching.

## Notes On Contributors

Poh Sun Goh, MBBS, FRCR, FAMS, MHPE, FAMEE, is an Associate Professor and Senior Consultant Radiologist at the Yong Loo Lin School of Medicine, National University of Singapore, and National University Hospital, Singapore. He is a graduate of the Maastricht MHPE program, a member of the AMEE TEL committee, and a Fellow of AMEE. ORCID: https://orcid.org/0000-0002-1531-2053

John Sandars is Professor of Medical Education at Edge Hill University Medical School, Ormskirk, UK, and is Co-Chair of the AMEE Technology Enhanced Learning Committee. ORCID: https://orcid.org/0000-0003-3930-387X

## Declarations

The author has declared the conflicts of interest below.

Both authors are members of the AMEE Technology Enhanced Learning Committee.

## Ethics Statement

This is an opinion piece incorporating a review of literature on educational scholarship and topic of digital scholarship.

## External Funding

This article has not had any External Funding
