# Supplementary material for: Digital Scholarship – rethinking educational scholarship in the digital world
Source: MedEdPublish (2016). 2019 Apr 15;8:85. [Version 1] doi: 10.15694/mep.2019.000085.1 (PMC10712586; doi:10.15694/mep.2019.000085.1)
Supplement: Peer review history

# Reviewer report: Reviewer response for version 1

**DOI:** 10.21956/mep.19818.r29847

**Published:** 2019-04-20

**Authors:** P Ravi Shankar

**Affiliations:** 1 American International Medical University

This review has been migrated. The reviewer awarded 4 stars out of 5 Thank you for the opportunity to review this interesting manuscript. With the increasing availability of online journals, periodicals and blogs the traditional journal is being increasingly seen as slow and bureaucratic. Many traditional journals are developing a significant online and web presence. Some have online blogs and commentaries supplementing the journal. Some journals allow for quick publication of findings often followed by post-publication peer review and discussions. MedEdPublish is a good example. The long peer-review process and often the delay in publishing accepted articles are drawback of traditional journals though many journals today offer online publication soon after acceptance. Social media will play an increasingly important role in medical education. There already exist devices which can record lectures and other activities and upload them on various cloud-based platforms and social media. Post-publication peer review may become increasingly common. Technology makes it easier for educators from resource-constrained settings to collaborate and conduct research. The article would be of interest to all medical educators.

Reviewer Expertise:

NA

No decision status is available

# Reviewer report: Reviewer response for version 1

**DOI:** 10.21956/mep.19818.r29848

**Published:** 2019-04-16

**Authors:** Ken Masters

**Affiliations:** 1 Sultan Qaboos University

This review has been migrated. The reviewer awarded 4 stars out of 5 A really very useful introduction to the concept of digital scholarship. The authors have begun with the background of educational scholarship, and have then reflected upon the impact of the “digital” part – the technology, and have broadly given an overview of three important areas: Open access publication and non-peer reviewed outputs, Social Media, and Analytics. I particularly like the fact that the authors have been able to introduce a necessarily technical subject with as little confusing technical jargon as possible. In addition, they have supported the concepts with useful and current references so that those readers interested in pursuing the topics further are easily able to do so.Finally, the authors have also nicely addressed some of the possible problems facing the digital scholar, as the innovative nature of technology inevitably brings with it new challenges.Two small issues:• “Almetrics” should be “Altmetric” (with a capital “A” and a “t” but no “s”) if referring to the site, or “altmetrics” (a small “a” and a “t”) if referring to the concept.• The current “Take-Home Messages” is a repeat of the Conclusion – these should rather be short and pithy bullet points. (I’m not sure if this was in error or done intentionally, but it does need to be rectified.).

Reviewer Expertise:

NA

No decision status is available
